# Environmental DNA Assay for the Detection of the American Bullfrog (*Lithobates catesbeianus*) in the Early Stages of the Invasion in the Ebre Delta

**DOI:** 10.3390/ani13040683

**Published:** 2023-02-15

**Authors:** Nuria Sanz, Nati Franch, Rosa-Maria Araguas, Jordi Viñas, Oriol Vidal

**Affiliations:** 1 Laboratori d’Ictiologia Genètica, Department of Biology, Faculty of Sciences, Universitat de Girona, C/Maria Aurèlia Capmany 40, 17003 Girona, Spain; 2 Parc Natural del Delta de l’Ebre, Av. Catalunya, 46, 43580 Deltebre, Spain

**Keywords:** American bullfrog, biological invasions, DNA barcoding, environmental DNA, invasive species, *Lithobates catesbeianus*

## Abstract

**Simple Summary:**

The introduction of alien species is one of the major causes of biodiversity loss. The American bullfrog (*Lithobates catesbeianus*) is considered to be one of the most harmful invasive species which overall threatens native amphibians. The detection of an invasive species in the first stage of its arrival is critical to control its colonization and to avoid its establishment. This early detection requires tools with high sensitivity. Methods based on the analysis of free environmental DNA (eDNA) are promising. The present article develops an eDNA assay to monitor the early process of invasion of the American bullfrog in the Ebre Delta (Spain), in a scenario where the presence of bullfrog specimens is really low and scarcely detected by traditional methods. In 2018, the first bullfrog tadpoles were found for the first time in the Ebre Delta. Two years after, despite the species not being well established, our eDNA assay detected the presence of bullfrogs in several locations. This methodology proved to yield a higher sensitivity with a lower sampling effort than traditional methods. Based on our experience, we also provided solutions to face challenges associated with the use of eDNA. Developing a rapid and low-cost-effective protocol to use in the early stages of an invasion (as occurred with the American bullfrog in the Iberian Peninsula) is essential to facilitate the detection, control, and eradication of an invasive species in the early stages of the invasion process.

**Abstract:**

The American bullfrog (*Lithobates catesbeianus*) is considered to be one of the most harmful invasive species. In the Iberian Peninsula, this species had been cited occasionally until the year 2018, when *L. catesbeianus* appeared in the Ebre Delta, and, for the first time, it started breeding in a territory of the Peninsula. Using environmental DNA (eDNA) analysis and visual surveys, the American bullfrog invasion in the Ebre Delta was monitored across two consecutive years (2019–2020). No specimens were observed in 2019, and results for the eDNA survey also failed to detect this species in the Delta. In 2020, two individuals were captured and, under the most conservative criteria to constrain the number of positive detections, eDNA analyses detected the presence of the American bullfrog in at least five locations. Performing an eDNA assay yielded a higher sensitivity with a lower sampling effort than traditional methods. Although the American bullfrog does not appear to still be well-established in the Ebre Delta, only a few bullfrog individuals could be enough for their establishment in suitable habitats. In this context, eDNA assays are essential tools to facilitate the detection, control, and eradication of this species in the first stage of the invasion process.

## 1. Introduction

The introduction of alien invasive species is considered to be one of the world’s major causes of biodiversity loss [1,2]. Freshwater ecosystems are especially susceptible to such introductions, and amphibians are probably the most fragile species in these ecosystems. Amphibians are globally threatened because of environmental changes, including climate oscillations, new diseases, and human exploitation of freshwater resources. Moreover, the dispersion of non-native frog species (especially *Discoglossus pictus* (Otth, 1837) and *Lithobates catesbianus*) may pose a significant threat to ecological equilibrium, while contributing to the new disease expansion and the displacement of native species [3].

The Iberian Peninsula is one of the richest herpetofauna areas of Europe, and more than 30 species of amphibians are found in the territories of Spain [4]. Within this territory, the protected area of the Ebre Delta comprises a large wetland with rice paddy fields in the mouth of the Ebre River, which hosts several amphibian species. The Ebre Delta is a key area for the conservation of freshwater biodiversity in the Mediterranean basin hotspot [5], and it represents a fragile ecosystem, endangered by climate change and several established invasive species [6,7]. Irrigation channels, sea passages, lagoons, and ponds set up an interconnected network that is difficult to monitor.

### 1.1. The American Bullfrog

The American bullfrog (*Lithobates catesbianus*) is native to Eastern North America and it is one of the world’s 100 worst invasive alien species in the Global Invasive Species Database [8]. It is also the most widely established alien amphibian species; it has been introduced in 59 regions throughout the world during the past two centuries [9]. American bullfrogs are large animals, reaching up to 900 g in weight and 20 cm in length. They may out-compete native species, acting as predators [10], or transmit exotic diseases such as *Batrachochytrium dendrobatidis* (*Bd* [11]) [12,13], and they have been responsible for the depletion and the extinction of many amphibian populations [14,15]. Both Spanish and European laws (Spanish Real Decreto 630/2013; UE Regulation 1143/2014) forbid the possession, transport, and trade of bullfrog specimens.

**Figure 1:**
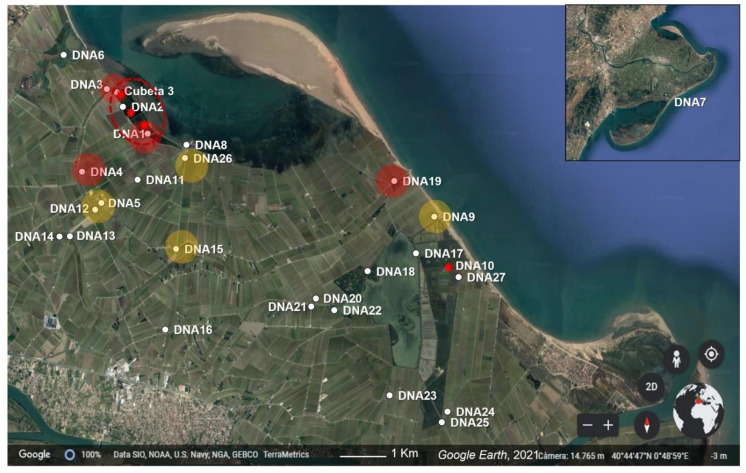
Map of the Ebre Delta with sampled locations. Samples where American bullfrog eDNA was confirmed by multiple primer sets are shaded in red. Samples where American bullfrog eDNA was confirmed by a single primer set are shaded in orange. Red symbol indicates the place where adult or juvenile bullfrog specimens were captured. Red circled area: ground zero.

In Europe, the American bullfrog has been introduced in several regions and it has naturalized populations in France, Germany, Italy, Belgium, and Greece [16]. In the three first countries, the depletion of native amphibian species has been documented [17]. In the Iberian Peninsula, some sporadic bullfrog records were reported at the end of the twentieth century, mostly related to escapes from closed farms. At the beginning of this century, some specimens were occasionally captured in the Collserola Mountains (Barcelona) and in the Canary Islands, the latter being suspected of introduction through trade [18,19]. However, all of these records reported non-successful introductions, and the study from Ficetola et al. [16] considered this species to be eradicated in the Peninsula. More recently, in 2009, bullfrog tadpoles were detected in Barcelona among aquarium trade from Italy [20] and in the Ebre Delta, where a single specimen was recorded in 2012 [21]. Until 2018, American bullfrog records in the Iberian Peninsula have been limited to these occasional sightings. Unfortunately, this situation changed in June of that year, when several tadpoles were reported in a lagoon of the Ebre Delta (Cubeta 3, herein ground zero, Figure 1) and the reproduction of the bullfrog in the Iberian Peninsula was confirmed for the first time.

### 1.2. Environmental DNA (eDNA) for Monitoring the Process of the Invasion

The process of biological invasion in freshwater ecosystems takes place in several stages (introduction, establishment, and spreading to the new habitat), which starts with the transport of the specimens from native or invaded areas to a new location [22]. The early detection of the species in the first stage of introduction is critical to control its colonization and to avoid its establishment and the subsequent expansion [23,24]. This detection needs to be reliable even when densities are very low, so that it can significantly increase the chances of eradication and reduce the economic and ecological impacts [25,26]. The effective management of early-stage invaders requires tools with high sensitivity, which are not always available for aquatic ecosystems, where traditional surveillance methods are not reliable indicators of occurrence [27]. In recent years, non-invasive methods based on the analysis of free environmental DNA (eDNA) have been developed [28]. This analysis is especially useful in aquatic ecosystems, both in freshwater and marine environments [29,30,31]. One of the big advantages of eDNA is the detection of extant populations with a very small number of individuals [32], as it allows for the early detection of biological invasions [24]. In amphibians, eDNA allows for the detection of larval stages of species with taxonomical identification problems, and DNA-barcoding protocols have been used to monitor populations [30,33,34].

The design of the eDNA barcoding assay is focused on targeting specific DNA regions that can be amplified with a conventional polymerase chain reaction (PCR), and can thus detect the organism. For the American bullfrog, previous eDNA analyses of water samples have demonstrated the usefulness of this technique [29,35,36]. The study by Ficetola et al. [29] proved the high sensitivity of the method with positive detections in natural lagoons of 1000–10,000 m^2^, where just one or two adult specimens had been observed during visual surveys. Dejean et al. [35] confirmed the validity of this method for species detection at very low densities, by reporting up to five times more positive sites than with using diurnal and nocturnal surveys. However, all of these previous studies comprised regions (south-west France) where the species had been introduced, where there were stable populations, and where its identification by eDNA could be contrasted by traditional (visual) methods.

Our study developed a rapid and low-cost-effective protocol based on the use of eDNA barcoding to monitor the early stages of the American bullfrog invasion in the Ebre Delta. It deals with a completely different scenario compared to other similar studies, as American bullfrog specimens have been rarely observed in the studied region. The analyses were launched in 2019 and continue up to the present. This study aimed to compare traditional and molecular methods of detection, to discuss the first eDNA positive results (2020 survey), and to explain the potential errors and challenges of the eDNA assay.

## 2. Material and Methods

### 2.1. Study Area and Bullfrog Detection

In June 2018, about 1000 tadpoles of the American bullfrog were found in three lagoons (DNA1, DNA2, and Cubeta 3 locations) in the north part of the Delta (northern hemidelta, Figure 1, Table 1), making this the first reported reproduction event of this species in a natural ecosystem of the Iberian Peninsula (Table 1).

Since then, the Delta Natural Park implemented a rigorous plan to monitor and eradicate the species, including tadpole coop traps, adult terrestrial traps, terrestrial transects, and acoustic surveys. Surveys were more intensive in the area where tadpoles were found and its surroundings, but also occurred all over the northern hemidelta. Coop traps (fyke nets) were placed during the entire reproductive period in areas where tadpoles and adults had been found, specifically where the environmental and physic-chemical conditions were more suitable to the bullfrog (eight traps in the ground zero area and six in the eDNA10 area). Traps were visited every three days. Adult terrestrial traps were built to capture adult bullfrogs specifically. They were made of metal mesh with bait inside, and with an only-entry door. Three adult traps were placed in the ground zero area. Terrestrial transects and acoustic surveys consisted of 17 routes previously designed along the north hemidelta, and were repeatedly made during the months of the American bullfrog activity. Acoustic surveys were made in several points of the routes for 15 min in the same point and between 22:00 and 24:00 o’clock, when the song of bullfrog males to attract females is usually listened to. (Table 1, and see details in chrome-extension://efaidnbmnnnibpcajpcglclefindmkaj/https://mediambient.gencat.cat/web/.content/home/ambits_dactuacio/patrimoni_natural/especies_exotiques_medinatural/llista_sp_catalogades/amfibis/granota-toro/Informe-granota-toro.-PNDE.-2020.pdf, accessed on 20 December 2022). About 1000 larvae were found in 2018 and at least three adult specimens were heard, all within ground zero. No specimens were found anywhere else. Fortunately, these three lagoons were artificial and water connection with the rest of the Delta could be easily locked. To contain the terrestrial expansion of the species, a six km metallic fence was built during July 2018 to isolate the ground zero area. After summer, some juvenile post-metamorphic individuals were captured (76), most within the fence but some just outside (Table 1). At this moment, the three lagoons were highly salinized with a lethal salt concentration (>30 g of salt/L) for all amphibians in the larval phase. Afterwards, no more specimens were seen nor heard in the Delta until June 2020, when two deceased specimens were found: one in the DNA10 site (Figure 1), located seven km from the ground zero, and another one found in ground zero (Cubeta 3), a few days after the last eDNA survey.

### 2.2. eDNA Sampling and Extraction

In June 2019, the first eDNA survey was performed in eight locations: seven close to the ground zero, where the species had been seen the year before (Figure 1), and an external location (DNA7) in the south of the Delta area (southern hemidelta). In 2020, two eDNA surveys were performed, in June and July, covering the whole breeding season of the American bullfrog. During the June survey, nine locations were sampled, eight already included in the 2019 survey (DNA3-9 and Cubeta 3), plus one more location close to the site where a new specimen was found in June 2020 (DNA10). In July, sampling was extended up to 23 locations distributed throughout the northern hemidelta. Sampling was designed according to results from previous surveys, in locations close to the ground zero area and to DNA10, and considering all locations that were connected with these areas via water channels (Figure 1). Environmental conditions did not differ between sites. In all cases, ponds were of still water with moderate turbidity (Supplementary Figure S1). Temperature and salinity ranges were 21.8–24.3 °C and 0.9–2.41 g/L, respectively, in June 2019, and 23.2–26.2 and 0.8–1.3 g/L, respectively, in July 2019.

For each eDNA survey, samples from all locations were collected the same day with in situ water filtration to avoid contamination [37]. All non-disposable sampling equipment was cleaned with a 10% dilution of bleach after their use. A filter funnel (250 mL and 47 mm; Thermo Fisher Scientific, Melbourne, Australia) with a peristaltic pump and cellulose nitrate filters of 0.45µm diameter were used. A total volume of 500 mL of water was filtered in each location, in two separate filters (250 mL each) to avoid clogging. Each filter was subsequently folded and preserved in a 1.5 ml tube with 1 ml of ATL lysis buffer (Qiagen, Hilden, Germany). Negative control samples (nuclease-free water) were filtered every five locations. In the lab, the extraction process continued with a digestion with 100 µL of proteinase K added to each filter in ATL and with overnight incubation with shaking at 120 rpm and 37 °C. DNA was subsequently purified using the DNeasy Blood & Tissue Kit of Qiagen, increasing the AL lysis buffer and ethanol volumes up to 500 µL. The protocol was modified so that all of the filtered volume for a single location was transferred to the same spin column. After the final elution, eDNA extractions were diluted to 1:10, 1:100, and 1:1000 with nuclease-free water. Negative controls were processed simultaneously to eDNA filtering volumes to monitor putative contaminations during the sampling and extraction processes. DNA extraction and the subsequent amplifications were performed in separate rooms. A positive control was obtained by extracting DNA from American bullfrog muscular tissue using the same DNeasy Blood & Tissue Kit of Qiagen.

### 2.3. eDNA Amplification

PCRs were first performed with primers cytbF1 and cytbR1 [29], which amplified a fragment of 79 base pairs (*bp*) of the cytochrome *b* mitochondrial gene (cytb) (Table 2). This set of primers was tested for the native amphibians living in the Ebre Delta, *Bufo bufo* (Linnaeus, 1758), *Pelophylax perezi* (López-Seoane, 1885), and *Lissotriton sp* (Bell, 1838), and for another alien species, the painted frog, *Discoglossus pictus,* and it yielded positive amplifications in *Discoglossus pictus*. This species is native to Mediterranean Africa and was introduced in France and north-east Catalonia [38]; therefore, its presence cannot be excluded in the Delta. Then, two additional American bullfrog primers (F2 and R2) were designed to be used in combination with the original ones. For this, the software Primer3 was used from multiple alignments of American bullfrog sequences in Geneious software, version 5.6 [39]. This new set of primers amplified a fragment of 200 *bp* of the cytb that included the region amplified by cytbF1 and cytbR1 and failed to be amplified in all native amphibians as well as in the painted frog. The basic local alignment search tool also showed that these primers do not match with high scores to any other sequences stored in GenBank. Then, amplifications of all primer combinations with these new primers (cytbF1 + cytbR2, cytbF2 + cytbR1) in tissue (muscle) and positive eDNA samples of American bullfrog were checked. The primer set cytbF1 + cytbR2 (150 *bp*) was selected as the optimal size combination for eDNA amplification, and cytbF2 + cytbR1 were kept as an alternative set.

The presence of native amphibians (*Bufo bufo*, *Epidalea calamita* (Laurenti, 1768), *Lissotriton helveticus* (Grigory Razumovsky, 1789), *Pelobates cultripes* (Cuvier, 1829) and *Pelophylax perezi*) is recorded all along the Delta [40], and hence in all sampled locations. Therefore, primers 16SA-L and 16SB-H, described in Vences et al. [41] as amphibian DNA barcoding, were used as positive controls to check for PCR inhibition in eDNA samples. Previously, the positive amplification with these primers was checked in tissue samples of the most abundant amphibian in the Delta (*Bufo bufo* and *Pelophylax perezi*). These primers amplified a fragment of 594 *bp* of the mitochondrial 16S rRNA gene and PCR conditions were the same as used by the American-bullfrog-specific PCRs (Table 2).

**Table 2:** Primers used in this study for universal amphibian and American bullfrog DNA amplification.

Universal Amphibian	Primer Sequences	References
16SA-L	CGCCTGTTTATCAAAAACAT	Vences et al. (2005) [41]
16SB-H	CCGGTCTGAACTCAGATCACGT	Vences et al. (2005) [41]
American bullfrog		
CytbF1	TGCCAACGGAGCATCATTC	Ficetola et al. (2008a) [29]
CytbF2	GTTAATAACGGCTGACTCCTA	This study
CytbR1	ATAAAGGTAGGAGCCGTAGT	Ficetola et al. (2008a) [29]
CytbR2	GATATTTGGCCCCATGGT	This study
Primer set	Fragment length	
CytbF1 + CytbR2	168 *bp*	
CytbF2 + CytbR1	111 *bp*	
CytbF1 + CytbR1	79 *bp*	
16SA-L + 16SB-H	594 *bp*	

For all primer combinations, PCRs had a total volume of 30µL with two μL of eDNA extraction, three μL buffer (BIOLINE) 10 × 0.15 μL of Taq DNA polymerase (BIOLINE) 5 u/μL, 0.6 μL primer forward 10 μM, 0.6 μL primer reverse 10 μM, three μL dNTP MIX two mM, and 0.9 μL MgCl_2_ 50 Mm. For all reactions, thermal cycling conditions consisted of an initial denaturation step at 94 °C for three min, followed by 10 cycles of touch-down of 94 °C for 30 s, 65–55 °C (−1 °C per cycle) for 1.5 min, and 72 °C for 1.5 min, and 30 cycles of 94 °C for 30 s, 55 °C for 1.5 min, and 72 °C for 1.5 min, plus a final step of 72 °C for five minutes.

All PCR reactions were conducted in a PCR UV chamber. In general, for each sampled location, two or three replicates of the 1:10 and the 1:100 dilutions of eDNA extraction were amplified with specific (cytbF1 + cytbR2) and universal primers (16SA-L + 16SB-H). Replicates of the original eDNA extraction (1:1) were also amplified for the 2020 survey samples. Additionally, for some locations of the 2020 survey, 1:1000 dilutions were also used. This distribution yielded at least 6 replicates per sample and per primer set. In all cases, negative controls of the extraction (filtered and processed nuclease-free water) and PCR negative controls, were included, as well as positive controls from a DNA extraction of American bullfrog tissue.

All PCR products were visualized in an electrophoresis gel at 2% agarose with GelRed^TM^ in an Axygen Gel system. Electrophoresis ran for 45 min to obtain a clear distinction between the small fragment amplified by specific bullfrog primers and primer dimers (Supplementary Figure S2).

### 2.4. Design of a Pipeline to Validate Results in Front of the Possibility of False Positives and Negatives

**Figure 2:**
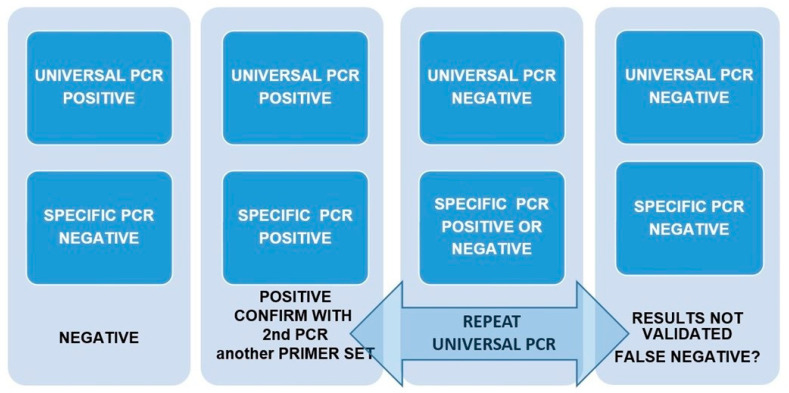
Pipeline designed to produce and validate results considering the amplifications of a bullfrog-specific PCR (specific PCR) and an amphibian universal PCR (universal PCR). Although not found in this study, if both universal and specific PCRs resulted in negative results, they could not be validated.

Based on the extremely low density of American bullfrog specimens in the studied region, a low detection probability of our eDNA PCR assay was assumed. Consequently, three initial replicates in three diluted concentrations were set up, summing up to eight replicates per sample. Following this basal design, a pipeline (Figure 2) to produce and validate the results considering the amplifications of a bullfrog-specific PCR (specific PCR) and an amphibian universal PCR (universal PCR) was established. If there was positive amplification in the universal PCR in at least two replicates but none of the eight replicates were amplified by the specific PCR, these samples were considered to be negative. Alternatively, if the universal PCR failed or when any of the eight replicates were amplified by the American bullfrog PCR, a second round of PCRs was performed to confirm results (Figure 2).

## 3. Results

### 3.1. 2019 Survey

Despite tripling the sampling effort from 2018, traditional methods (trapping and visual and acoustic tracking) failed to detect the American bullfrog inside and outside of the ground zero area.

Universal amphibian primers (16SA-L + 16SB-H) were amplified in at least two out of six replicates in all sampled locations, discarding the presence of PCR inhibitors that could lead to false negative results in the specific amplifications (cytbF1 + cytbR2). No amplification of bullfrog DNA was observed in any water sample at any dilution in these 2019 samples.

### 3.2. 2020 Surveys

One deceased specimen was found in the DNA10 location in June 2020, and sampling via traditional methods was intensified close to this location, in ground zero and surrounding areas. This increased effort detected only one other deceased individual in Cubeta 3 (ground zero) four days after the eDNA survey, and another individual was also heard in Cubeta 3, in a acoustic survey at the end of July.

#### 3.2.1. June eDNA Survey

Universal amphibian primers (16SA-L + 16SB-H) were not amplified in the 1:1 eDNA extractions, but the amplification was positive in at least two out of the six replicates of the 1:10 and 1:100 dilutions in eight locations (all locations except DNA4, DNA5, and DNA9). For these three sites, a second round of PCRs was performed, excluding the 1:1 eDNA extractions and including three replicates of the 1:1000 dilution. This second round of amplification was positive in two to five out of six replicates per sample.

Initially, American-bullfrog-specific PCRs were amplified only in one replicate for the 1:100 dilutions in the DNA9 sample (Table 3). Then, a second round of PCRs was performed for all of the locations, including three replicates of the 1:1000 dilutions. Amplification was then successful for three replicates in DNA5 and four in DNA9 (Figure 1). The overall amplification success in these samples for the June survey was then 3/16 in DNA5 and 5/16 in DNA9 (Table 3).

#### 3.2.2. July eDNA Survey

Samples from the second survey (July 2020) had two–three PCR replicates of the 1:10, 1:100, and 1:1000 dilutions, with a total of eight replicates per sample (Table 3). Universal amphibian amplification was successful in all 23 samples with two to five positive replicates per sample, except in DNA22, DNA23, and DNA26. A second round of universal PCRs performed for these three locations yielded positive amplifications in four to five replicates per sample.

American bullfrog eDNA was successfully amplified in eight (out of the twenty-three) locations, with one to five positive replicates per sample. A second round of PCRs confirmed bullfrog eDNA in four of these eight positive locations, plus in two more locations (DNA5 and DNA9). However, cross-contamination in a negative control of the first round of the cytbF1 + cytbR2 PCR was detected. These results were excluded and the alternative primer set (cytbF2 + cytbR1) was used to confirm all positive results. The cytbF2 + cytbR1 combination amplified American bullfrog eDNA in five out of the twenty-three samples, which were also amplified in the first or second round of the PCR with the primer set cytbF1 + cytbR2 (Table 3).

In summary, the eDNA survey indicated the presence of the American bullfrog in 10 (at least one positive PCR) out of 23 locations. However, in some of the July 2020 samples (DNA1, 3, 9, and 19), bullfrog eDNA was only detected in one replicate, despite DNA9 being positive (5/16) in the June survey, and bullfrog detection was confirmed in DNA1, 3, and 19 samples with the primer set cytbF1 + cytbR2. In five of the ten positive samples (DNA1, 3, 4, 19, and Cubeta 3) amplifications of the American bullfrog, eDNA was positive with both tested primer sets (Table 3).

## 4. Discussion

### 4.1. Challenges of eDNA to Track Invasive Species

Because traditional surveys only detected the species occasionally in only one or two locations in the Delta, it is clear from our results that eDNA reported higher sensibility with a low catch effort. eDNA-based methods to monitor invasive species in aquatic ecosystems have been designed and applied successfully to detect these species even at very low densities [35,42,43]. In our study, this methodology allowed us to infer a more complete picture of the extent of the invasion process in the first stage, as has been reported in other previous studies [29,35,44]. Moreover, the logistical requirement for eDNA sampling and the persistence of eDNA in the environment beyond the presence of the species are also arguments strengthening the use of eDNA methods. However, similarly to other monitoring methods, eDNA methodology also has several critical points along the whole process, from sampling to the interpretation of the data [45].

#### 4.1.1. Environmental DNA Capture

A first challenge of eDNA assays is water filtration with cellulose nitrate filters. Filters with small pore size are strongly recommended for eDNA sampling as they optimize eDNA capture at low concentrations [37,46]. However, in turbid water bodies with a lot of organic matter or suspended sediment, filters clog quickly and the filtering rate is so slow that it is impossible to filter an optimal volume (at least 500 mL), which is especially required when eDNA is scarce. Several alternatives have been suggested: increasing pore size, pre-filtering samples, or reducing the water volume of samples. All of them lead to lower yields of target DNA [37,47]. Alternatively, Hunter et al. [47] increased the filtered volume and obtained a higher DNA yield by combining several filters in a single Phenol-Chloroform-Isoamyl DNA extraction. This alternative solution was adapted by using the DNeasy Blood & Tissue Kit of Qiagen (Hilden, Germany), which is recommended for eDNA [48]. The total desired volume was filtered using as many filters as necessary (two in our case) which were then preserved and processed separately until the eDNA was transferred to the spin columns of the kit. Thus, the digestion volume of the two filters belonging to the same location was collected in a single DNeasy mini spin column. This modification in the DNA extraction protocol allowed us to recover the eDNA from a total 500 mL volume, avoiding problems of filter clogging.

#### 4.1.2. PCR and False Positive/Negative Results

The design of primers may be related to false positive and false negative results of the eDNA protocols. In this sense, the length of the fragment might be a critical issue. On the one hand, if the fragment is very short, the risk of amplification artefacts and sporadic contamination (both causing false positive results) is higher. On the other hand, very long DNA sequences are prone to false negatives as long templates do not persist in the environment [49]. Therefore, most published papers with water sampling have diagnostic PCR fragment sizes shorter than 150 *bp* [35,50,51].

Although we took precautions to reduce the risk of contamination (DNA extraction in a separate room, the PCR in a UV-chamber, and amplification of negative and positive controls), cross-contamination detected in some negative controls made us exclude some ‘positive’ results and use alternative primer sets for the specific PCR.

False positive results could also be due to the persistence of eDNA when the species had already disappeared from the water body. Dejean et al. [35] proved that bullfrog eDNA persisted in freshwater ecosystems for a maximum of two weeks after animal removal.

Nevertheless, the main problem to face was to avoid false negatives by the presence of PCR inhibitors. This is particularly concerning when sampling turbid waters such as those from wetlands in the Ebre Delta. Several protocols have been proposed to improve eDNA yield, such as adding chemical compounds or performing mechanical processes to remove inhibitors during DNA extraction [47,52]. An alternative solution is the dilution of eDNA extractions to reduce inhibitors. This easy method does not have the economic cost of removing potential PCR inhibitors. However, this approach can be problematic when DNA concentrations are low, because diluting the extractions also reduces DNA concentration and hence the sensitivity of the PCR assays [52,53]. The negative effect of inhibitors can also be assessed using a second PCR with universal primers. In our case, a set of unspecific amphibian primers (16SA-L and 16SB-H) was used to amplify all eDNA extractions. As other species of amphibians (mainly *Pelophylax perezi*) were expected in all of the sampled locations, negative results were indicative of inhibition. Our positive results from a universal PCR show that dilution avoided inhibitor effects in all of the samples. Previous results from McKee et al. [52] show that a 10-fold dilution is enough to reduce qPCR inhibition effectively. In most of our samples, a 1:100 dilution was necessary to avoid the effects of inhibitory compounds. As discussed just below, this 1:100 dilution did not compromise the overall sensitivity of the PCR assay because several replicates were simultaneously amplified (at least two for each dilution). Therefore, the possibility of false negatives due to the random and unequal distribution of the very few DNA molecules in the dilutions was avoided.

#### 4.1.3. Replicates and Threshold of Positivity

The last critical point when using eDNA is the suitable number of replicates and the threshold of positive tests to consider the presence of the species to be certain. The detection of alien invasive species relying solely on DNA-based methods has been controversial, especially when such detection can result in costly management implications [45]. In this context, performing an optimal number of replicates to avoid missed detections and setting the minimum number of positive replicates to avoid false positives are both strictly necessary. To assess these parameters, previous studies have calculated the detection probabilities of eDNA analyses. However, this is only possible when eDNA results can be compared with traditional methods of detection outcomes or with experiments in controlled conditions [29,35]. In our case, since the eDNA studies started, visual and acoustic surveys detected only a couple of specimens in a very localized area. Therefore, the detection probability of our PCR assay could be compared to traditional methods but it was not able to be tested empirically.

In general, the detection probability is not high when the density of the species is low. Ficetola et al. [54] recommend at least eight PCR replicates to avoid false negatives when the detection probability is lower than 0.5. Goldberg et al. [53] conducted controlled experiments with different densities of the invasive New Zealand mudsnail (*Potamopyrgus antipodarum*) and used three replicates for each density treatment to reach the detection of even one individual in 1.5 L of water. In these scenarios with several replicates per sample, it is important to consider the possibility of crossed or sporadic contamination. Thus, to avoid false positives, Taberlet et al. [55] recorded an allele only if it was observed in at least two out of ten replicates when they analyzed samples with little DNA. More recently, Ficetola et al. [54] suggested the same strategy in eDNA metabarcoding studies, remarking that a sufficient number of replicates was necessary to avoid false negatives with low detection probabilities.

According to the pipeline described in methods to validate results and considering all of the replicates together, 14–16 specific PCRs per location were performed in the 2020 survey. Of these 16 amplifications, negative results in most of the 1:10 dilutions suggested the presence of PCR inhibitors in this dilution. Accordingly, replicates of 1:10 dilutions were abandoned and the analysis was performed with 12 replicates instead. Following the recommendations from previous studies [54], positive identification was made when the specific PCR amplified at least two of the twelve probes. Under these criteria, there were two positive locations in the June 2020 survey and seven in the July survey.

Moreover, Sepulveda et al. [56] suggested using different PCRs targeting different genomic locations. This would increase the reliability of positive detections. In our case, an alternative primer set of the specific PCR (cytbF2 + cytbR1) was used to confirm positive amplifications of American bullfrog eDNA in samples of the most recent survey (July 2020). This approach confirmed the detection of the American bullfrog in five locations (Table 3).

### 4.2. Early Invasion of the American Bullfrog in the Ebre Delta Revealed by eDNA

According to the arguments discussed so far, several restrictions should be and have been applied in the interpretation of the undertaken American bullfrog eDNA assay in the Ebre Delta. Even under the most conservative scenario, the presence of this species in at least five locations can be confirmed in the last survey (July 2020, Figure 1). The detection of the species in several locations through eDNA analyses contrasts with a very local detection of only two individuals through visual or acoustic surveys. This suggests an early first stage of the invasive process [24], and it shows once again that eDNA assays improved detection sensibility with respect to the traditional methods, with a much lower sampling effort [35].

Interestingly, the highest number of positive replicates was found in the ground zero area, where the first bullfrog tadpoles were observed in June 2018 (Table 1 and Figure 1). However, the eDNA sampling of the 2019 year was negative, and the presence of this species in the Delta was not reported again until June 2020, in a place seven kilometers from ground zero. In 2020, a single deceased specimen was found in the DNA10 location, and the eDNA analyses confirmed the incipient introduction of this species again. Curiously, the eDNA survey of June 2020 failed to detect the species in the DNA10 location, but it was detected in DNA9, where waters from the DNA10 region were collected. Within the ground zero area, a single deceased specimen was found and another individual was heard in July 2020, after the last eDNA survey (Table 1). These history records and the eDNA results might suggest two alternative hypotheses regarding the American bullfrog invasion in the Ebre Delta. First, it is possible that the eradication plan carried out in 2018 in the ground zero area was sufficient to eliminate tadpoles, but some post-metamorphic terrestrial individuals survived and escaped from this area before the construction of the metallic fence was completed (end of July 2018). Then, if few individuals survived but they were not established, the bullfrog density in 2019 could have not been enough to be detected even by eDNA analyses (either because the concentration of eDNA was too low or because the sampling was not extensive enough). Detections in 2020 should then be attributed to these survivor individuals. The concentration of positive detections in the ground zero area in the 2020 survey could be suggesting that two years after the first introduction, the invasive American bullfrog persisted in the first site of detection. Alternatively, it could be possible that at least some individuals came back to the original site of introduction to reproduce. The fact that 29 post-metamorphic juveniles (>200 g weight) were captured in autumn 2018 outside of the fence surrounding the ground zero area supports this hypothesis. The second invasion focus that seemed to appear in 2020, close to the place where a dead specimen was found, could have had an origin in individuals that spread out from the ground zero area.

Alternatively, it is possible that the 2018 eradication plan was successful and that the American bullfrog was eradicated. Therefore, the two specimens found and the positive eDNA results in 2020 would correspond to a new invasion process, or possibly multiple introductions in the two invasion focuses. In other countries, reiterated intentional releases of the American bullfrog have been documented [16,57], and the same pattern could be taking place in the Ebre Delta. European legislation prohibits introductions of the American bullfrog and its commercial farming is completely forbidden in the Iberian Peninsula (Royal Decree 630/2013, 2 August). However, this legislation could change if the species was already established. For instance, the Autonomous Government of Catalonia (SRM/1/2019, 17 May 2019) has changed its law and recently allowed the commercialization of the blue crab *Callinectes sapidus* (Rathbun, 1896), another invasive species in the Ebre Delta [58]. Therefore, a hypothetical premeditated release of bullfrogs could be related to the gastronomic and economic potential of the American bullfrog commercialization and the possibility of a law change if the species is successfully introduced.

The failed attempts of American bullfrog establishment in the Delta should not lower our guard. Blackburn et al. [59] link the success of the three stages of the invasion process (introduction, establishment, and spread) to several critical aspects. Specifically, the number of invasive specimens plays an important role in the first stage of the invasion, because a larger number of transported individuals will increase the probability of success. The number of arrived individuals at the second stage is also important, as a small number of individuals leads to reduced genetic diversity that can compromise the process of adaptation to the new habitat. For the American bullfrog, Ficetola et al. [60] suggested that an extremely low number of founder specimens can be enough for a successful invasion process. For instance, it is estimated that the Italian invasive population descended only from two females and one male introduced in 1930. The success at the second stage also requires that the new habitat has similar conditions to those of the native one [61]. Ficetola et al. [62] suggests that certain environmental factors (mainly related to the climate) are critical to determine the probability of the establishment of the American bullfrog. The projection of the environmental suitability for the bullfrog made by these authors (see Figure 3 in [62]) indicates the south and the west of the Iberian Peninsula as being less suitable for bullfrog establishment. This could explain that despite some individuals having been reported in these regions, they have never become invasive [18,19,62]. However, the situation is quite different in the northeast (including the Ebre Delta region), where the environmental suitability for bullfrog establishment reaches up to 50/100, according to Ficetola et al. [62].

The monitoring of the American bullfrog in the Ebre Delta through classical and molecular tools is expected to continue in the upcoming years, at least for some years after both of the methodologies fail to detect the species. We have started extending sampling to never-surveyed areas and the preliminary results were negative, but more of a sampling effort is still necessary.

## 5. Conclusions

Our eDNA analyses allowed us to delimitate the extent of the invasion of the American bullfrog in the Delta, yielding a higher sensitivity with a lower sampling effort than traditional methods. In this context, eDNA assays are essential tools to facilitate the detection, control, and eradication of the species in the first stage of the invasion process in the Ebre Delta. Even at a low population density, the American bullfrog may represent a high level of risk for the conservation of biodiversity in the Ebre Delta ecosystem, a fragile ecosystem already endangered by climate change and the establishment of other invasive species [58,63,64]. In such a situation, Darling and Mahon [45] stated that, despite controversial arguments, DNA-based methods might be the only tool to promote management actions prior to the assumption of unacceptable invasion risks.

## Figures and Tables

**Table 1:** Capture effort and results via traditional methods. Sampling effort: for terrestrial transects, it was measured as the total number of travels along transects. For acoustic surveys, it was the number of 15 min stops made to listen to bullfrogs along all of the travels. For tadpole trapping, it was the total number of visits to the coop traps. For adult traps, it was the total number of visits to the traps. Captures are the number of tadpoles or juvenile individuals captured or listened to. All tadpoles and juvenile individuals were captured within the ground zero except for one deceased individual found 7 km from the ground zero area in June 2020.

	2018			2019			2020		
	Sampling Effort	Number of Captures	Period	Sampling Effort	Number of Captures	Period	Sampling Effort	Number of Captures	Period
Terrestrial transects	73	76	August–November	257	0	January–November	195	2	April–October
Acoustic surveys	15	3	June–July	80	0	April–November	39	1	June–August
Tadpole coop traps	235	1057	June–October	792	0	May–September	704	0	May–October
Adult traps	-			90	0	July–September	150	0	June–October
eDNA	-			8	0	July	32	10	June–July

**Table 3:** Number of positive replicates for the specific amplification of American bullfrog eDNA with respect to the total number of replicates, in samples from the 2020 surveys. NEG: no amplification. The number of replicates for each tested dilution (1:10, 1:100, 1:1000) is indicated between parenthesis. F1 + R2 means that PCRs were performed with the cytbF1 and cytbR2 primer set. F2 + R1 means that PCRs were performed with the alternative set of primers cytbF2 + cytbR1. All specific amplifications were validated with positivity in the amplifications with amphibian universal primers in the first or second round (samples that were amplified at the second round via universal PCRs are indicated with an asterisk (*)). Note: In the June survey, a second round of the specific PCR was performed also in DNA10, because it was in this location that the adult individual was found. In the July survey, we included DNA9 in the second round of the specific PCR because it resulted in a positive in the June survey.

	Total Positives/Total Number of Replicates		First Round of PCR F1 + R2			Second Round of PCR F1 + R2			Alternative PCR F2 + R1		
June survey	F1 + R2			1:10 (3)	1:100 (3)	1:10 (2)	1:100 (3)	1:1000 (3)			
DNA3	NEG			NEG	NEG						
DNA4*	NEG			NEG	NEG	NEG	NEG	NEG			
DNA5*	3/14			NEG	NEG	NEG	2/3	1/3			
DNA6	NEG			NEG	NEG						
DNA7	NEG			NEG	NEG						
DNA8	NEG			NEG	NEG	NEG	NEG	NEG			
DNA9*	5/14			NEG	1/3	1/2	2/3	1/3			
DNA10	NEG			NEG	NEG	NEG	NEG	NEG			
July survey	F1 + R2	F2 + R1	1:10 (3)	1:100 (3)	1:1000 (2)	1:10 (2)	1:100 (3)	1:1000 (3)	1:10 (2)	1:100 (3)	1:1000 (3)
DNA1	1/16	1/8	NEG	NEG	1/2	NEG	NEG	NEG	NEG	NEG	1/3
DNA3	1/16	2/8	NEG	1/3	NEG	NEG	NEG	NEG	NEG	NEG	2/3
DNA4	4/16	2/8	NEG	NEG	1/2	NEG	1/3	2/3	NEG	NEG	2/3
DNA5	5/16	NEG	NEG	NEG	NEG	NEG	3/3	2/3	NEG	NEG	NEG
DNA6			NEG	NEG	NEG						
DNA7			NEG	NEG	NEG						
DNA8			NEG	NEG	NEG						
DNA9	1/16	NEG	NEG	NEG	NEG	NEG	1/3	NEG	NEG	NEG	NEG
DNA10			NEG	NEG	NEG						
DNA11	NEG		NEG	NEG	NEG						
DNA12	2/16	NEG	NEG	NEG	1/2	NEG	NEG	1/3	NEG	NEG	NEG
DNA13	NEG		NEG	NEG	NEG						
DNA14	NEG		NEG	NEG	NEG						
DNA15	2/16	NEG	NEG	1/3	1/2	NEG	NEG	NEG	NEG	NEG	NEG
DNA16	NEG		NEG	NEG	NEG						
DNA17	NEG		NEG	NEG	NEG						
DNA18	NEG		NEG	NEG	NEG						
DNA19	2/16	1/8	NEG	NEG	1/2	NEG	1/3	NEG	NEG	1/3	NEG
DNA20	NEG		NEG	NEG	NEG						
DNA21	NEG		NEG	NEG	NEG						
DNA22*	NEG	NEG	NEG	NEG	NEG	NEG	NEG	NEG	NEG	NEG	NEG
DNA23*	NEG		NEG	NEG	NEG	NEG	NEG	NEG	NEG	NEG	NEG
DNA24	NEG		NEG	NEG	NEG						
DNA25	NEG		NEG	NEG	NEG						
DNA26*	2/16	NEG	NEG	1/3	1/2	NEG	NEG	NEG	NEG	NEG	NEG
DNA27	NEG		NEG	NEG	NEG						
cubeta3	6/16	5/8	3/3	2/3	NEG	NEG	1/3	NEG	NEG	3/3	2/3

## Data Availability

All raw data will be freely available upon request to the authors.

## Supplementary Materials

The following supporting information can be downloaded at: https://www.mdpi.com/article/10.3390/ani13040683/s1, Figure S1: Picture of one of the sampling locations in the Ebre Delta; Figure S2: Example of an agarose gel of American bullfrog PCR with positive locations (DNA1, 3 and 4). Negative control of extraction (-), positive control (+), molecular weight (PM) and negative control of PCR (-) are included. Note: bands with the most quick mobility correspond to primer dimers and they appear everywhere but in the positive control.
