# Supplementary material for: Environmental DNA Assay for the Detection of the American Bullfrog (Lithobates catesbeianus) in the Early Stages of the Invasion in the Ebre Delta
Source: Animals (Basel). 2023 Feb 15;13(4):683. doi: 10.3390/ani13040683 (PMC9952411; doi:10.3390/ani13040683)
Supplement: Supplementary file 1 [file animals-13-00683-s001.zip › animals-2146289-supplementary.pdf]

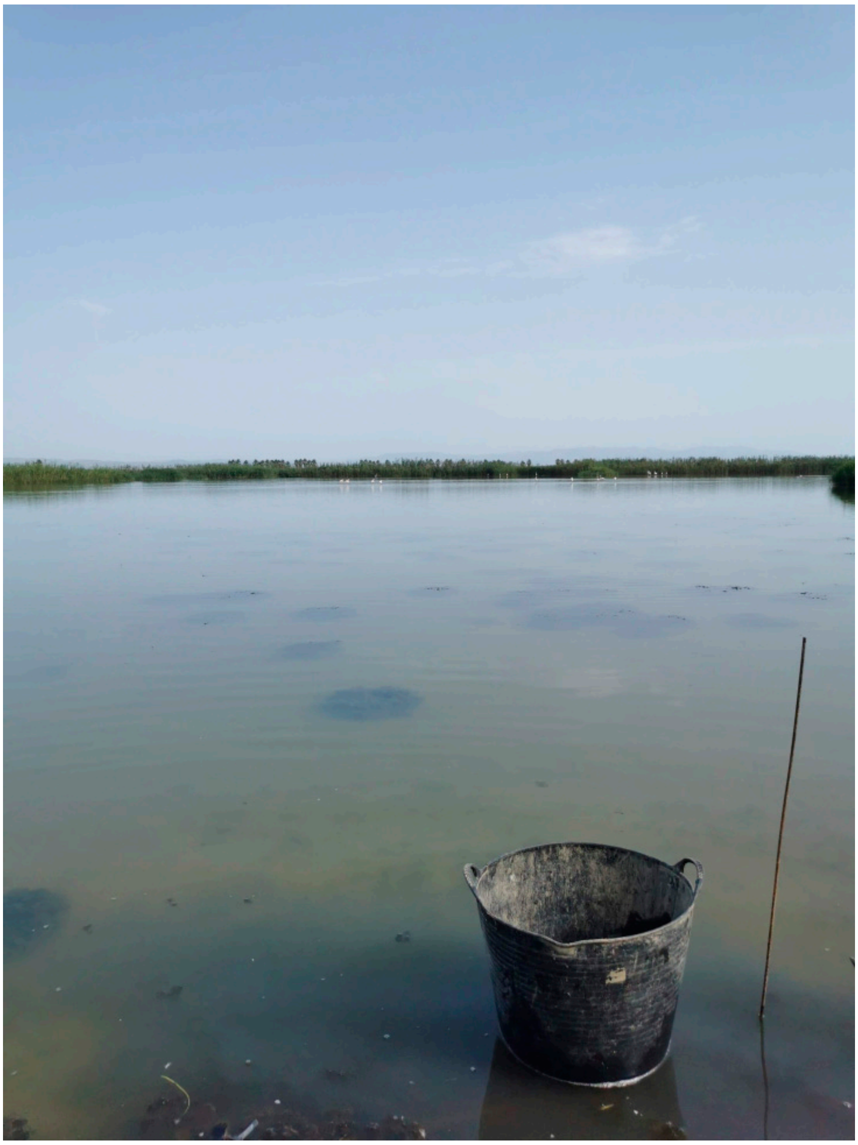

Figure S1. Picture of one of the sampling locations in the Ebre Delta.

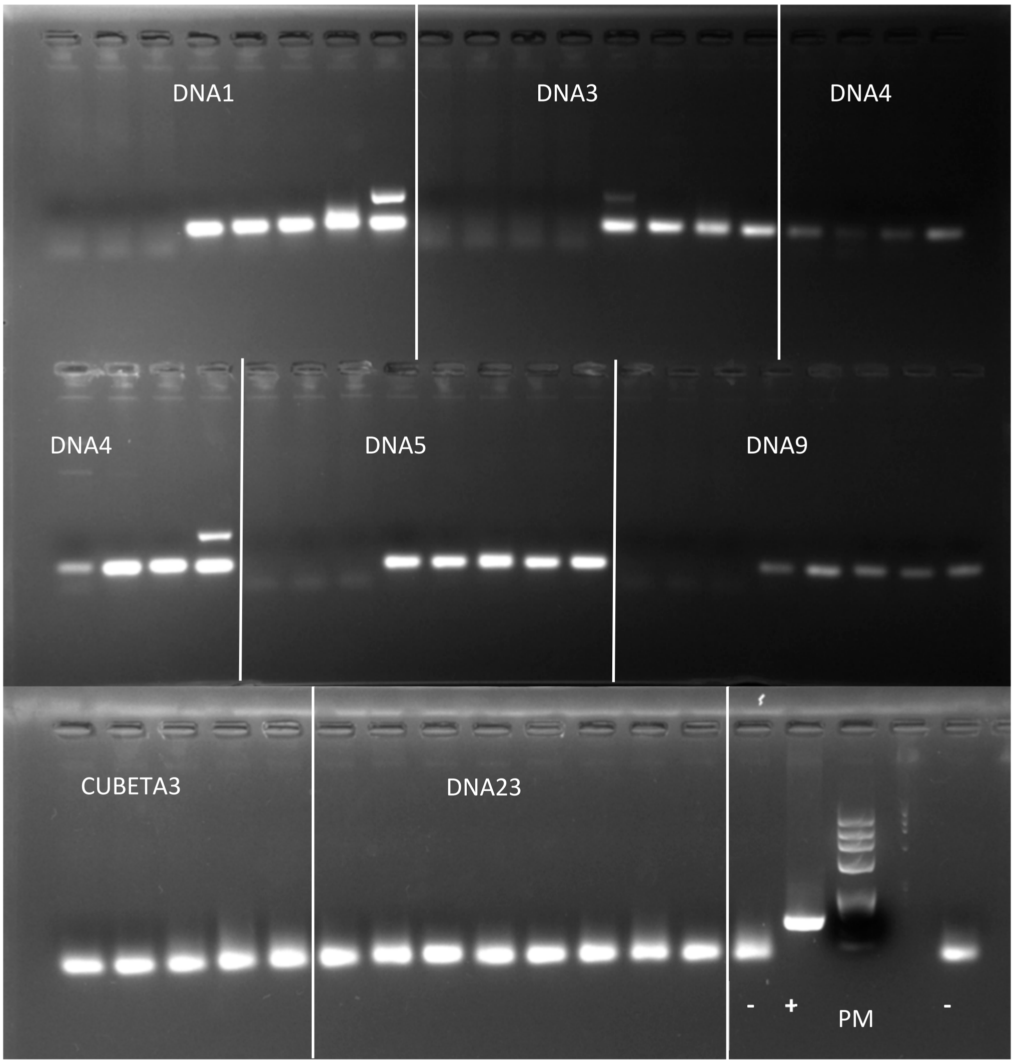

Figure S2. Example of an agarose gel of American bullfrog PCR with positive locations (DNA1, 3 and 4). Negative control of extraction (-), positive control (+), molecular weight (PM) and negative control of PCR (-) are included. Note: bands with the most quick mobility correspond to primer dimers and they appear everywhere but in the positive control.
